# Molecular markers for tolerance of European ash (*Fraxinus excelsior*) to dieback disease identified using Associative Transcriptomics

**DOI:** 10.1038/srep19335

**Published:** 2016-01-13

**Authors:** Andrea L. Harper, Lea Vig McKinney, Lene Rostgaard Nielsen, Lenka Havlickova, Yi Li, Martin Trick, Fiona Fraser, Lihong Wang, Alison Fellgett, Elizabeth S. A. Sollars, Sophie H. Janacek, J. Allan Downie, Richard. J. A. Buggs, Erik Dahl Kjær, Ian Bancroft

**Affiliations:** 1 Department of Biology, University of York, York, UK; 2 Department of Geosciences and Natural Resource Management, University of Copenhagen, Denmark; 3 Computational and Systems Biology, John Innes Centre, Norwich, UK; 4 Department of Crop Genetics, John Innes Centre, Norwich, UK; 5 School of Biological and Chemical Sciences, Queen Mary University of London, London, UK; 6 The Genome Analysis Centre, Norwich, UK; 7 Department of Molecular Microbiology, John Innes Centre, Norwich, UK

## Abstract

Tree disease epidemics are a global problem, impacting food security, biodiversity and national economies. The potential for conservation and breeding in trees is hampered by complex genomes and long lifecycles, with most species lacking genomic resources. The European Ash tree *Fraxinus excelsior* is being devastated by the fungal pathogen *Hymenoscyphus fraxineus,* which causes ash dieback disease. Taking this system as an example and utilizing Associative Transcriptomics for the first time in a plant pathology study, we discovered gene sequence and gene expression variants across a genetic diversity panel scored for disease symptoms and identified markers strongly associated with canopy damage in infected trees. Using these markers we predicted phenotypes in a test panel of unrelated trees, successfully identifying individuals with a low level of susceptibility to the disease. Co-expression analysis suggested that pre-priming of defence responses may underlie reduced susceptibility to ash dieback.

Many tree species are particularly badly affected by biotic stresses caused by introduced pathogens and insect parasites. In recent decades, many parts of the world have experienced the devastating results of these epidemics, which have impacted trees of great economic and/or ecological importance^1^. American Chestnut Blight, transmitted by the wind-borne ascospores of the fungus *Cryphonectria parasitica*, is thought to have been accidentally introduced to the Eastern US in the 1900s from Asia. By 1940, it had effectively wiped out the species from its native range^2^. Dutch Elm disease, which is caused by infection from one of several species of the ascomycete genus *Ophiostoma* transmitted by the elm bark beetle, has wreaked havoc on the native populations of elm trees across America, New Zealand and Europe since its identification in 1921^3^. *Musa* species (banana and plantains) are susceptible to black sigatoka disease, caused by the ascomycete fungus *Mycosphaerella fijiensis.* This has had a huge impact on global banana and plantain production and export, with countries such as Grenada losing their plantations completely^4^. Anthracnose fungi (usually *Colletotrichum* or *Gloeosporium* species) are a global problem, attacking many hardwood tree species^5^. Fusiform rust is a fungal (*Cronartiium quercuum* f. sp. *fusiforme*) disease affecting Southern pines, causing timber growers to lose millions of dollars annually. These and many other fungal pathogens are having a serious impact on the future of many economically and ecologically important tree species.

A chronic tree disease known as Ash Dieback is caused by the fungal pathogen *Hymenoscyphus fraxineus.* First recorded in Poland^6^, it infects European Ash (*Fraxinus excelsior*) and has led to huge losses across Europe. It is transmitted by wind-borne spores that colonise healthy leaves in the summer months, before advancing along the petiole into the branches of the tree. The fungus remains in the petioles after the leaves have dropped, releasing spores the following summer, initiating a new infection cycle. Over a period of several years, most infected trees will exhibit progressive crown dieback and necrotic lesions that can lead to the death of the tree and damage to the timber.

In common with many tree species, the genetics of *Fraxinus excelsior* had, until very recently, been little studied. Production of bi-parental mapping populations in species with such long generation times means that linkage and QTL mapping can take decades. Genome sequencing of *F. excelsior* began in 2012 upon the arrival of Ash Dieback in the UK, and the first draft genome sequences have now been released (www.ashgenome.org; http://oadb.tsl.ac.uk), but ordered assemblies of genomic resources can also take years to produce. In order to meet challenges in species such as *F. excelsior* in a timely manner, new genetic tools are required.

Transcriptome-based methods are becoming more commonly applied for genetic analysis. By focussing only on transcribed sequences, the complexity associated with sequencing large or polyploid genomes is reduced, enabling gene orders to be more rapidly inferred^7^,^8^. In addition, transcript quantification can enable differential and co-expression analyses^9^,^10^,^11^,^12^.

Associative Transcriptomics is a potent new method for rapidly identifying molecular markers associated with trait variation across a diversity panel, exploiting both gene sequence variation and gene expression variation, and can be effective even where advanced genomics resources are not available^6^. With a suitable panel of diverse accessions, resolution is such that high quality tightly-linked markers, and in some cases candidate genes, can be identified with relative ease^7^,^13^. Like most association genetics methods, this identification is made more efficient through ordering of the genes and markers in genome order, as loci can be identified through clustering of independent markers within the range of linkage disequilibrium (LD). Gene order can be gleaned from fully assembled genome information for the species of interest, or inferred from genome collinearity with a closely-related species (where available). Many tree species however, are only distantly related to herbaceous or tree species with suitable genome data, making gene order inference all but impossible. Researchers wishing to analyse genetic components of trait variation in trees will often find themselves beginning with, at best, largely unordered genomic sequence scaffolds, or may have to produce *de novo* assemblies. Nevertheless, even unordered genome sequence scaffolds represent a suitable resource for the development of gene models to use as reference sequences onto which mRNA-seq reads can be mapped for the identification of SNPs and quantification of gene transcript abundance, together comprising a functional genotype.

## Results

In this study, we aimed to identify markers for low susceptibility to ash dieback via Associative Transcriptomics, even though this approach had not previously been used to identify markers associated with disease susceptibility in plants. We used a panel of Danish ash trees that varied from completely healthy specimens to individuals that suffered severely from ash dieback. The trees were selected in 2012–13 from amongst a large number of trees in Denmark, where symptoms of the ash dieback disease have been widespread since 2005. A total of 182 trees were used as a training panel for the development of the association models (Supplementary dataset 1) and 58 further trees were used as a test panel for validation of predictive capability.

In order for the training panel to include specimens with a wide range of susceptibility to ash dieback, it was necessary to select trees originating from diverse sites across Denmark. It was not possible to assess all of the accessions in a controlled trial, but in an attempt to avoid potential inaccuracies associated with phenotyping trees in such variable environments, putatively unrelated trees were chosen if they had been scored across multiple years and/or multiple test environments. In addition, to detect and correct for inconsistencies between the field sites, a subset of genotypes from each site was also assessed in an experimental nursery. In contrast to the training panel, the test panel subsequently used for validating markers associated with dieback damage consisted of a set of trees grown under exactly the same conditions (same site, same age, same disease pressure, grown from seedling in the same nursery), and all expected to be unrelated to the genotypes in the training panel.

Several grafts were made for each accession using uninfected material, and then whole leaf samples were collected from each successful ramet as soon as they flushed. The leaves from each accession were pooled to produce a single sample for RNA extraction before sequencing with 100 bp Illumina HiSeq reads. These sequence reads were aligned against a reference composed of 41,521 unigenes from the British Ash Tree Genome Project (www.ashgenome.org/transcriptomes). Gene models were constructed with the CLC Transcript Discovery Plugin (QIAGEN Bioinformatics, Aarhus, Denmark), using Illumina HiSeq transcriptome data from the British Ash Tree Genome Project mapped to the BATG_0.4 reference genome. Unigenes were extracted by selecting the longest transcript per gene. The susceptibility to ash dieback of the tree sequenced in this project is unknown.

After mapping mRNA-seq reads from the 182 tree training diversity panel to the unigene reference sequences, transcript abundance was calculated as reads per kilobase per million aligned reads (RPKM), and 470,494 SNPs were detected as described previously^14^. Population structure in the panel was inferred by using the kernel-PCA and optimisation method, PSIKO^15^, which revealed two main population clusters. The two clusters did not correlate with the geographical locations of the mature trees, but more likely reflect the distribution of specific breeding materials throughout the country (Supplementary dataset 1).

Following filtering to remove SNPs with minor allele frequencies < 0.05 and genes with transcript abundance mean RPKM < 0.4, 174,346 SNP markers and 32,441 gene expression markers (GEMs) remained that were suitable for Associative Transcriptomics. Along with a Q matrix produced by PSIKO, the SNP markers and mean damage scores for the years 2013 and 2014 were used in a mixed linear association model (MLM) following kinship assessment in the program TASSEL^16^. The same phenotype data were also used in linear regressions with the GEM markers, also incorporating the Q matrix as an additional fixed effect, in R (cran.r-project.org). The results are plotted in Supplementary Fig. 1. A single SNP and 13 GEMs passed the significance threshold following Bonferroni correction (alpha = 0.05).

According to the BLAST annotations, several of the gene models with the best associations with disease susceptibility are MADS box transcription factors. As the SNP and GEM association results should be independent, it is unlikely that so many related genes should be identified amongst the top results by chance. Rather, the results indicate SNP and GEM markers within a regulatory network of candidate genes that have a function in reducing the susceptibility to *H. fraxineus*. The top SNP (Supplementary Fig. 2; Gene_22343_Predicted_mRNA_scaffold3139), and top two GEM markers (Supplementary Fig. 3; Gene_19216_Predicted_mRNA_scaffold2427 and Gene_23247_Predicted_mRNA_scaffold3380) (all of which pass Bonferroni multiple test correction), which are also members of the MADS box transcription factor family based on their BLAST hits, were converted into conventional PCR markers for the SNP assay, and qRT-PCR markers for GEM assays.

As an indication of how predictive these markers would be, we performed a set of “take one out” permutations. Each of the accessions in the association panel was removed in turn, and the regressions repeated. A robust prediction marker would be one that appears frequently in the top results for each analysis, and indeed the markers selected for further analysis were always the top markers in each analysis. The two marker types were able to significantly predict the phenotype of the missing accessions either alone (SNP: R^2^ = 0.21, p = 1.2 × 10^−10^, GEM: R^2^ = 0.28, p = 1.4 × 10^−12^) or combined (R^2^ = 0.28, p = 1.4 × 10^−14^). We would expect that the three markers will be able to predict the phenotype of other accessions with a similar level of success.

**Figure 1:**
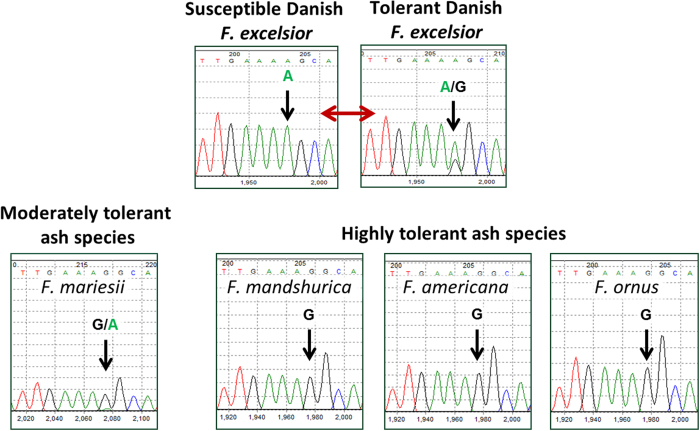
Sequence chromatograms showing the cSNP position in a number of Fraxinus species. Gene_22343_Predicted_mRNA_scaffold3139:2378 was assayed by PCR and Sanger sequencing directly from the cDNA of both susceptible and tolerant Danish *F. excelsior* accessions, as well as the moderately tolerant species *F. mariesii*, and the extremely tolerant species *F. mandshurica*, *F. americana* and *F. ornus*. The “G” base is either not present at all in *F. excelsior*, or at a low level compared to the “A” base. In contrast, the “G” base is prevalent in the more resistant species, with only a low level of the “A” base being detected in the moderately tolerant *F. mariesii*.

For the development of predictive PCR-based marker assays, both SNP and GEM marker types were assayed using cDNA produced from RNA purified from leaves. Assays were developed for the SNP marker showing the most significant association with the trait (Gene_22343_Predicted_mRNA_scaffold3139) and for the two most significant GEM associations (Gene_19216_Predicted_mRNA_scaffold2427 and Gene_23247_Predicted_mRNA_scaffold3380). Gene expression values based on qRT-PCR assays correlated well with the mRNA-seq-derived RPKM results for both of the GEM markers (R^2^ = 0.81 and 0.89, respectively; p < 0.001). For the SNP marker, as illustrated in Fig. 1, the allele associated with increased disease susceptibility is an A base, whereas the allele for reduced susceptibility (R, denoting a mixture of both G and A base calls) shows variable proportions of the two bases in the capillary sequencing traces. This characteristic is consistent with differential expression of alleles, so we refer to this type of marker as a cDNA-based SNP, or cSNP.

To establish if the GEMs and cSNP are indeed predictive for tolerance to the disease, leaves from a test panel of 58 additional Danish *F. excelsior* accessions were sampled, and the new accessions assayed for the three markers. We also tested four *Fraxinus* species known to have little or no susceptibility to the disease for comparison. In order to make predictions using the qRT-PCR results, it was necessary to standardise and rescale the qRT-PCR quantifications based on the spread of the RPKM quantifications from the original training panel. These rescaled values were then fitted to the regression line from their respective GEM association results to enable calculation of trait predictions for each of the 58 members of the test panel. The cSNP marker was simply genotyped based on the presence of the G peak in the sequence trace files, and then assigning the appropriate allele effect based on the output from the GWAS analysis performed in TASSEL. The predicted trait values were then compared to the damage scores recorded for the test panel in the summer of 2014. We established that both GEM markers were weakly predictive on their own (p < 0.05), and the cSNP marker was a moderately good predictor of the level of canopy damage (p < 0.01).

As complex traits such as disease tolerance are likely to be additive, we combined these markers into a single prediction to see if this increased the predictive power. In order to combine predictions of the differing marker types, the individual predictions were ranked, and the rank scores standardized. In this way, the mean and standard deviation of each of the individual marker effects will be the same, ensuring each marker will have an equal contribution to a combined prediction. Mean scores for each of the three standardized rank scores was taken to provide a combined rank score for each accession. These combined rank scores were then rescaled to a more intuitive value between 0 and 100 (representing predicted canopy damage score), which could then be correlated with the observed damage scores. This combined marker score was highly predictive of the observed damage (R^2^ = 0.24, p < 0.001) (Supplementary Fig. 4 and Supplementary dataset 2).

To further test the potential predictive capability of the markers, they were assayed in four *Fraxinus* species that show very low susceptibility to ash dieback: *F. mariesii*, *F. americana*, *F. ornus*, and *F. mandshurica*. The results were consistent with the observed phenotype, i.e. the GEM-based predictions were for low susceptibility and the cSNP marker showed that all of these species had entirely or predominantly the G base, as shown in Fig. 1.

As well as association studies, the mRNA-seq datasets can be used to undertake Weighted Gene Co-expression Network Analysis (WGCNA) (Supplementary Fig. 5; Supplementary dataset 3), which revealed a single module highly correlated with canopy damage (indianred4; R = 0.482, p = 3.39 × 10^−11^). This module contains 56 genes, exhibiting both positive and negative relationships with canopy damage, including the top two GEMs from the association analysis, which are highly connected. In fact, based on BLAST similarity, there are up to 10 additional MADS-box genes represented in this module. The MADS box gene *SVP* (Gene_24384_Predicted_mRNA_scaffold3716) as well as being involved in the flowering pathway, has been shown to be necessary for induction of Age-Related Resistance (ARR) response in *A. thaliana*^17^. The close relation of *SVP* in tomato known as *JOINTLESS* (Gene_22343_Predicted_mRNA_scaffold3139 and Gene_25477_Predicted_mRNA_scaffold4068) is involved in formation of abscission zones^18^. Other module members are also implicated in pathogen responses in *A. thaliana*, such as a protein kinase involved in mediating resistance to fungi (*ATRLCKV1_A3*; AT5G65530.1; Gene_15720_Predicted_mRNA_scaffold1694), a putative MATE transport protein that regulates plant disease resistance (*ADP1*; AT4G29140.1; Gene_15967_Predicted_mRNA_scaffold1872), and one of the “hub” genes in the network is related to a pathogenesis-related transcription factor (RAP2.7; AT2G28550.2; Gene_11904_Predicted_mRNA_scaffold1222)^19^,^20^,^21^. This module enrichment suggests that the cSNP and GEMs may be markers for pre-induction of pathogen resistance responses, providing greater tolerance. Such genetics-based priming could be analogous to induced priming^22^.

## Discussion

Associative Transcriptomics is widely applicable to plant species, requiring relatively little by way of pre-existing genomics resources. It focuses on transcribed sequences, meaning that the technology is not only able to identify gene sequence variants, but crucially for the disease study undertaken here also identifies gene expression variants. By identifying markers associated with the level of ash dieback damage observed in a diversity panel of trees, we were able to identify molecular markers that are capable of predicting low susceptibility to the disease. Significantly, these markers were identified from uninfected leaves and so probably reflect a mechanism different from induced disease resistance. It remains to be seen whether this form of disease tolerance is more stable than classical induced resistance; the observation that the same markers were present in other species of ash with very low susceptibility indicates this may be the case. The expression data analysed in a WGCNA provided clues as to the mechanism responsible for variation in disease tolerance.

With tree diseases becoming an increasing concern all over the world, there is a need for new technologies to assist breeding programmes. Time is of the essence if dead trees are to be replaced with less susceptible genotypes before the losses to industry and the ecosystem are irreversible, and predictive power is especially important in management of tree diseases where symptom development can take several years and may vary among natural habitats. We demonstrate here that by using Associative Transcriptomics, such problems are not intractable, and verifiable predictive markers for disease tolerance can be identified. We believe that the approach pioneered here could be used as a paradigm for identifying genetic markers in other tree disease epidemics. New strategies for reforestation can be designed once predictive molecular markers are available. For example, instead of breeding from a very small number of highly tolerant trees (which would lead to lack of genetic diversity), a wide range of genetically diverse individuals can be identified that are predicted to be tolerant. Use of such a panel for breeding will maintain background genetic diversity (essential for response to the next pest or disease challenge) whilst rapidly building numbers of resistant trees.

## Materials and Methods

### Selection of trees and identification of ash dieback phenotypes

The ash trees used in this study were selected in 2012 and 2013 in Denmark ten years after the first observations of ash dieback were reported^23^. The majority of ash trees from the selected stands were severely diseased and trees with varying levels of susceptibility were identified.

Association panel: A total of 213 trees were used for the development of the model and these trees originated from 4 groups selected in 2012. ‘Randers group’: 44 trees were selected at age 10 based on their damage level in a progeny field trial located at Randers (56.50 N, 10.04 E), which includes approximately 2,200 trees monitored for infection level since 2008. Details on their genetic origin and on the trial site are presented in Kjær *et al.*^24^. ‘Hørsholm group’: 20 trees selected at age 14 in a progeny field trial located at Hørsholm (55°86′ N, 12°48′ E) among approximately 600 tested trees monitored for infection level since age 11. Details on genetic origin and trial site are presented in Lobo *et al.*^25^.’ CSO group’: 19 trees were selected among 39 vegetatively propagated clones based on the average damage level at two clonal test sites in Denmark. Details on genetic origin and trial sites are presented McKinney *et al.*^23^. ‘Forest group’ consisted of 99 trees selected in Danish forests (2012 and 2013) across the country from heavily infected areas. 23 of these trees were selected as trees with heavy damage levels, while the remaining 96 of the trees were selected as mature healthy trees. The healthy trees were preferably chosen in areas where neighboring trees were heavily affected to ensure that high levels of infection pressure was present in the forest stand. All sampled trees for the training panel were putatively unrelated.

Scions were collected from the trees during January and February 2013 and grafted onto rootstocks of European ash seedlings and placed in a greenhouse until time of flushing and leaf sampling for RNA extraction. At least 7 additional grafts were made of each of the 96 symptom free forest trees (‘Forest group’) and planted in an experimental nursery in Hørsholm where they were subjected to natural infection pressure. This was done in order verify that their low levels of symptoms in the forests were not simply a result of disease escape since they did not originate from a common field trial. 18 of the trees selected from the trials sites (CSO, Randers, Hørsholm) were also propagated and planted for comparison in order to allow correction for effects of differences in damage level among the sites. The phenotypes in terms of damage level were characterized using a 7 step scoring scale where class 0 presents symptom free trees, 1:1–10% symptomatic crown damage, 2:10–25% crown damage, 3:26–50% crown damage, 4:51–75% crown damage, 5:76–99% crown damage, 6:100% crown damage (dead). Phenotypic values were reported as interval averages (0%, 5%, 12.5%, 37.5%, 62.5%, 62.5%, 87.5%, 100%) that were also used for calculation of average values across replications. The number of scoring classes were in some cases reduced to 5 steps by merging score2 + score3 and score 4 + score5 + score6 in order to make the scoring suitable for small trees. The phenotypes of the selected trees in the ‘Randers’ and ‘Scion’ groups were based on inspection of the trees in June 2012 (at time of selection) while the phenotype of trees in the ‘CSO’ group were based on the average damage level across all replications of the clones assessed June 2014 at both test sites. The phenotypes of the unhealthy trees belonging to the ‘forest group’ were assigned based on visual inspection at the time of selection. The trees selected as healthy trees in the ‘forest group’ were phenotyped based on the average damage level of the clonal replication planted in the experimental nursery assessed in June 2014. Finally, the phenotypic values were corrected by comparing the performance of the 18 trees originating from the field trials also planted in the experimental nursery. The correction for the effects of age and sites were important for the 20 trees from the ‘CSO group’ that were level corrected with -33% damage compared to the level in the nursery, while corrections for ‘Scion’ and ‘Randers’ were only 0%, and 3%, respectively. The resulting distribution of corrected phenotypes of the trees in the training panel is presented in Supplementary dataset 1.

Prediction Panel: 58 trees were selected for verification of the associations. These trees were selected at age 11 in the Randers trials (56.50 N, 10.04 E) in families not already sampled for the association panel and therefore all putatively unrelated to association panel trees. The trees were selected based on the average of their 2013 and 2014 health scoring with the objective to sample trees with both high and low susceptibility. However, since the disease had already created a high level of mortality (70%) in 2014, the sampling has probably not included the most susceptible genotypes since these are likely to have already been killed at the time of selection. Leaves for RNA extraction were sampled directly from the 66 trees in the field trial in May 2014.

### RNA extraction

For the association panel, whole leaves at flushing stage 3–5 were pooled from multiple ramets for each of 182 accessions (Supplementary dataset 1) and frozen in liquid nitrogen. Single leaf samples were harvested from each accession in the same way for the prediction panel. Samples were ground using a pestle and mortar in liquid nitrogen before RNA extraction using the standard protocol for the Omega Biotek E.Z.N.A Total RNA kit.

### SNP calling and transcript abundance quantification

Illumina sequencing and quality checking were performed as previously described^14^. Sequence reads were aligned to a unigene reference derived from the British Ash Tree Genome project (www.ashgenome.org/). The genes were functionally annotated with the best search result after local BLASTX searches in the curated Swissprot database downloaded from NCBI (http://www.ncbi.nlm.nih.gov/). SNPs were called by the meta-analysis of alignments as described in Bancroft *et al.*^12^, of mRNA-seq reads obtained from each of 182 ash accessions (Supplementary dataset 4). SNP positions were excluded if they did not have a read depth in excess of 20, a base call quality above Q20, missing data below 0.25, and three alleles or fewer. An additional noise threshold was employed to reduce the effect of sequencing errors, whereby ambiguous bases were only allowed to be called if both bases were present at 0.15 or above. Transcript abundance was quantified and normalized as reads per kb per million aligned reads (RPKM) (Supplementary dataset 5).

### Association analysis

The SNP dataset for the 182 accessions with mean damage scores for the 2013–2014 seasons was entered into the program PSIKO to assess the population structure and produce a Q matrix^15^.

The damage scores, PSIKO Q matrix, and SNP data for the 182 accessions was entered into the program TASSEL V3.0^16^. Minor allele states below 0.05 were removed from the SNP dataset leaving 174,346 SNPs (Supplementary data file 4), and a kinship (K) matrix was calculated to estimate the pairwise relatedness between individuals. These datasets were entered into a Mixed Linear Model (MLM) with optimum compression and P3D variance component estimation to decrease computing time for the large dataset (see Supplementary dataset 6 for results).

Gene expression associations were calculated by fixed effect linear model using R (http://www.R-project.org/) with RPKM values and the Q matrix inferred by PSIKO as the explanatory variables and damage score the response variable. R^2^ and significance values were calculated for each gene (Supplementary dataset 7).

### Marker prediction power estimates

The predictive power of the best GEMs and SNP were assessed using a “take one out” approach whereby each accession is removed from the SNP or GEM analysis in turn, and the association analysis performed for a given trait. The phenotype of the missing accession is then estimated based on the mean regression coefficients for the top 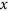 markers. In this way, the predictive power can be gauged *in silico* by the accuracy of the estimated to the observed trait data, and the robustness of each individual marker by the frequency that it appears in the top 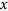 results. For this study, the power estimates were based only on the two GEMs and the single SNP that were selected for predicting phenotype of the test panel so that the prediction power estimates could be compared directly with the accuracy of the predictions in the test panel. These analyses were performed in R. To enable automating of the SNP prediction, a script was created incorporating the compressed mixed linear model^26^ implemented in the GAPIT R package^27^. The GEM prediction was performed with an adapted script used for the above GEM analysis.

### Damage Predictions

The damage scores for an additional panel of 58 trees were predicted. RNA was extracted as before, and cDNA synthesised using 1 μg of total RNA and the ImProm-II Reverse Transcription System (Promega, Madison, WI) according to manufacturer’s protocol.

For the SNP predictions, the cSNP Gene_22343_Predicted_mRNA_scaffold3139:2378 was scored from cDNA using the following primers: AshRB_22343-F1, GGTTTCTCTTCTGCAGCGAG; AshRB_22343-R3, TCCATGATCATCTTGCTGAG. Sanger sequences were obtained using the forward primer and sequence chromatograms viewed and the accessions exhibiting the less susceptible “R” (mixed A/G base) allele identified using Softgenetics Mutation Surveyor software. The accessions were then assigned an allele effect score according to the allele effects recorded for this marker in the original association analysis. In this case, ± 16.1293, with negative scores assigned if accessions possessed the “R” allele. These values could then be correlated with the observed phenotypes for these accessions.

To predict the phenotype of the 58 additional accessions using the GEMs, gene expression was scored by qRT-PCR using primers designed for Gene_19216_Predicted_mRNA_scaffold2427 (GTCGAGGAGGATGGTCAGTCAT, AATCTTGCGGAGGACCTATCG), Gene_23247_Predicted_mRNA_scaffold3380 (AGGGCAAGGCTTGGAAACAT TAGGCTTTTTTCTAGCTGCTTGTCA) and a GAPDH reference Gene_386_Predicted_mRNA_scaffold6 (CTGGGATCGCTCTTAGCAAGA, CGATCAAATCAATCACACGAGAA). qRT-PCR reactions were performed with SYBR Green fluorescence detection in a qPCR thermal cycler (ViiA^TM^ 7, Applied Biosystems, San Francisco, CA) using optical grade 384-well plates, allowing all reactions to be performed simultaneously for each target gene. Each reaction was prepared using 3 μl from a 2 ng/μl dilution of cDNA derived from the RT reaction, 5 μl of SYBR® Green PCR Master Mix (Applied Biosystems®), 200 nM forward and reverse primers, in a total volume of 10 μl. The cycling conditions were: 2 min at 50 °C, 10 min at 95 °C, followed by 40 cycles of 95 °C for 15 sec and 60 °C for 1 min with the final dissociation at 95 °C for 15 sec, 60 °C for 1 min and 95 °C for 15 sec. Three technical replicates were used for quantification analysis. Melting curve analysis was performed to evaluate the presence of non-specific PCR products and primer dimers. The specificity and uniqueness of the primers and the amplicons were verified by amplicon sequencing. The results were exported as raw data, and the LinRegPCR software^28^ was used for baseline correction. The resulting means of triplicate N_O_-values, representing initial concentrations of a target and reference genes were used to analyse gene expression. The qRT-PCR quantifications were re-scaled according to the mean and standard deviation of the RPKM values for the GEMs in the original association panel, and compared with the observed trait values for the prediction panel.

In order to create a combined prediction, the prediction scores for the cSNP and the GEMs were ranked, and to ensure equal contribution to the prediction for both marker types, the ranked values were then standardized. The mean of these standardized ranks was then rescaled between 0 and 100 (the minimum and maximum possible damage score) to make the predictions more intuitive. The combined prediction was then correlated with the observed damage scores.

### WGCNA

Weighted gene co-expression network was analysed as described by Zhang^26^ and Langfelder^29^. The R scripts and tutorials are available from the website http://www.genetics.ucla.edu/labs/horvath/CoexpressionNetwork/Rpackages/WGCNA/Tutorials/index.html. 182 accessions were clustered according to the RPKM values for 41,521 gene models. First, the samples were clustered, and 13 outlier accessions removed. Following re-clustering, the function pickSoftThreshold was used to identify the appropriate power β = 5 to raise the correlations to approximate scale-free topology within the network. The function blockwiseModules was then used to construct an unsigned network and identify modules with minModuleSize set to 10 and a mergeCutHeight of 0.25. Each module is summarised using the first principal component of gene expression, this value is termed the “module eigengene” (ME) which is the most representative expression pattern within the group of genes. We then correlated the summary profile (eigengene) for each module with the trait of interest.

## Additional Information

**How to cite this article**: Harper, A. L. *et al.* Molecular markers for tolerance of European ash (*Fraxinus excelsior*) to dieback disease identified using Associative Transcriptomics. *Sci. Rep.* **6**, 19335; doi: 10.1038/srep19335 (2016).

## Supplementary Material

Supplementary Figures

Supplementary Dataset 1

Supplementary Dataset 2

Supplementary Dataset 3

Supplementary Dataset 4

Supplementary Dataset 5

Supplementary Dataset 6

Supplementary Dataset 7
