# Supplementary material for: Molecular markers for tolerance of European ash (Fraxinus excelsior) to dieback disease identified using Associative Transcriptomics
Source: Sci Rep. 2016 Jan 13;6:19335. doi: 10.1038/srep19335 (PMC4725942; doi:10.1038/srep19335)
Supplement: Supplementary Figures [file srep19335-s1.pdf]

Molecular markers for tolerance of European ash (Fraxinus excelsior) to dieback disease identified using Associative Transcriptomics

Andrea L. Harper, Lea Vig McKinney, Lene Rostgaard Nielsen, Lenka Havlickova, Yi Li, Martin Trick, Fiona Fraser, Lihong Wang, Alison Fellgett, Elizabeth S. A. Sollars, Sophie H. Janacek, J. Allan Downie, Richard. J. A. Buggs, Erik Dahl Kjær, Ian Bancroft

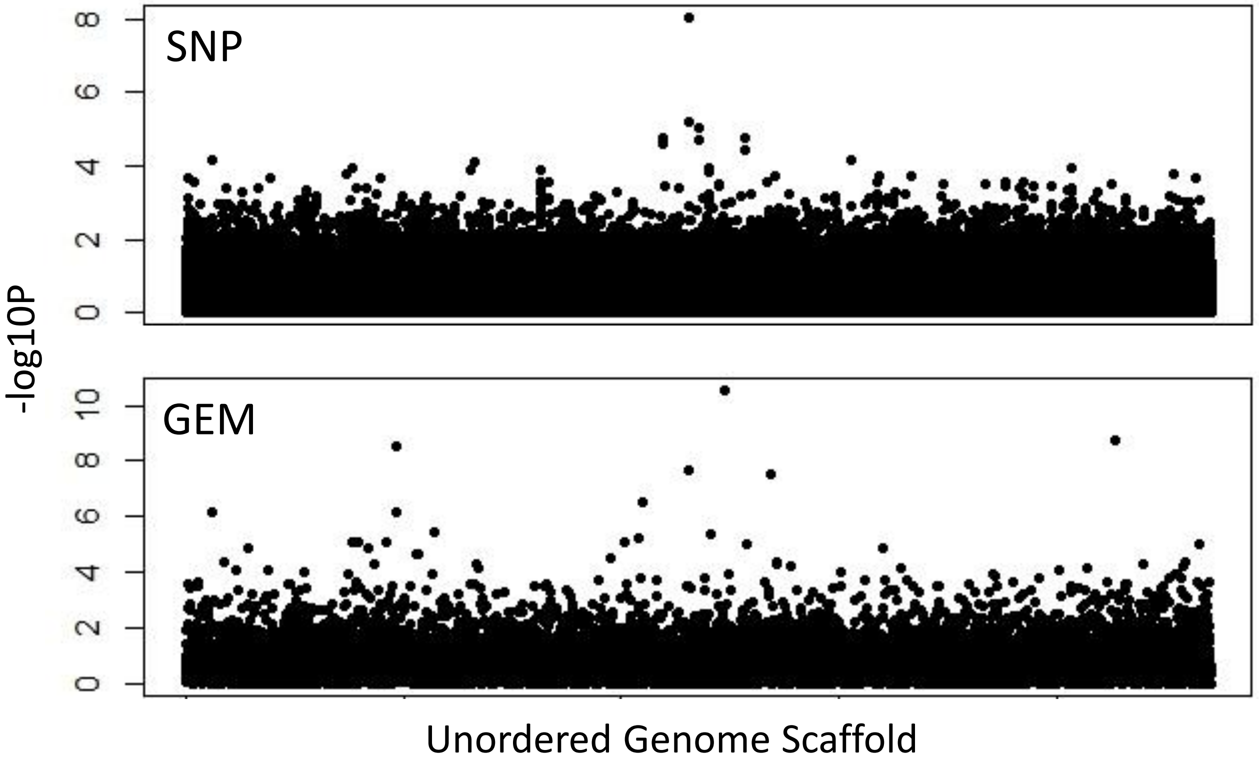

Supplementary Figure 1. SNP and GEM associations. Each point is a -log10 transformed P value for a single marker, presented according to their position within unordered genome scaffolds. Arbitrary scaffold ordering is the same on both the SNP and GEM plots.

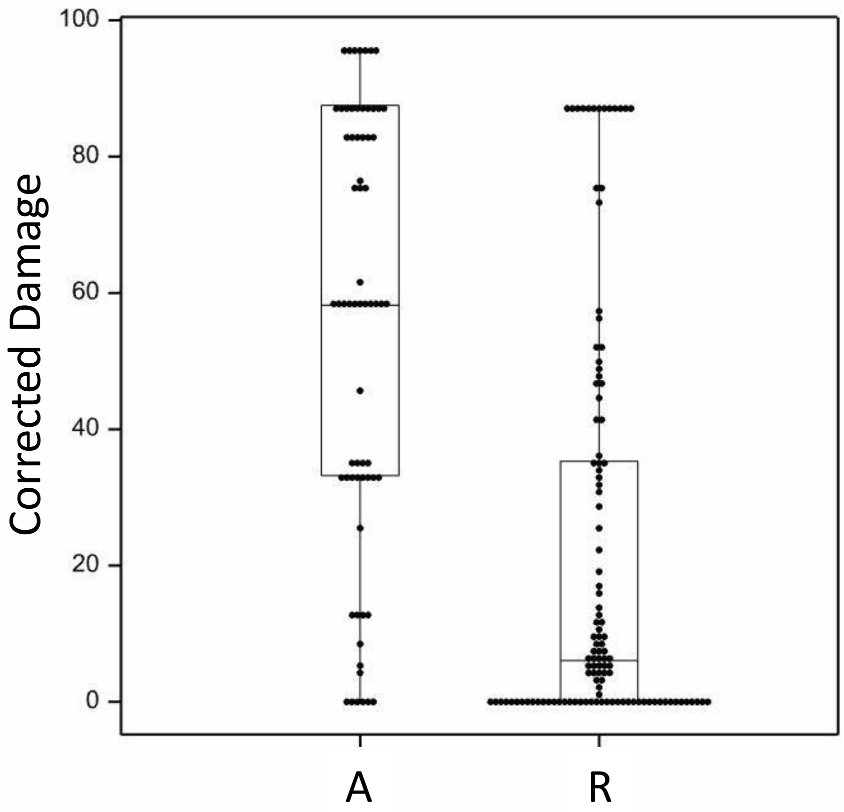

Supplementary Figure 2. Box and dot plot of cSNP alleles vs the corrected damage score. Each dot represents one of the 182 individuals in the association panel with damage scores and mRNA-seq base calls for marker Gene_22343_Predicted_mRNA_scaffold3139:2378. Only the A and R (A/G mixed base) alleles were observed in this panel.

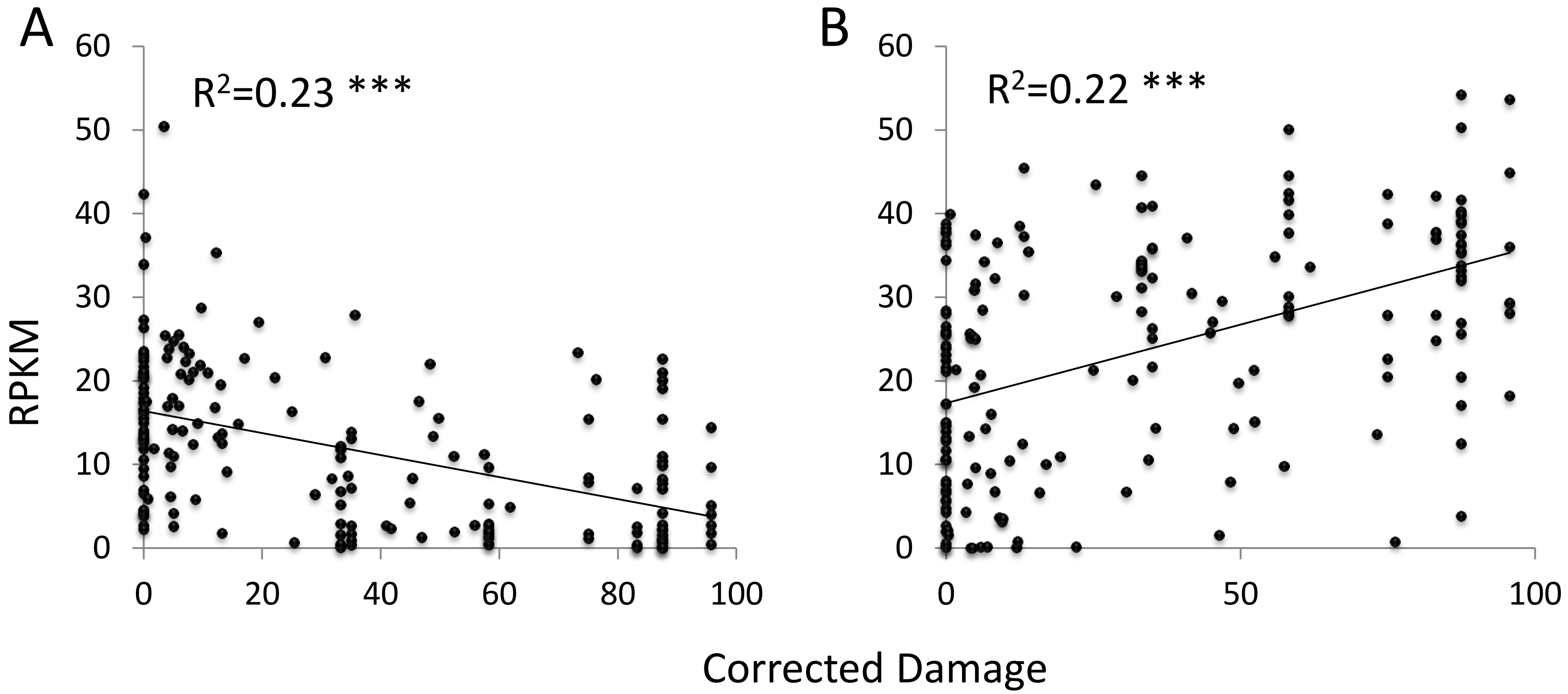

Supplementary Figure 3. Transcript abundance (RPKM) vs the corrected damage score for two GEMs; (A) Gene_19216_Predicted_mRNA_scaffold2427, and
(B) Gene_23247_Predicted_mRNA_scaffold3380. * P<0.05, ** P<0.01, *** P<0.001.

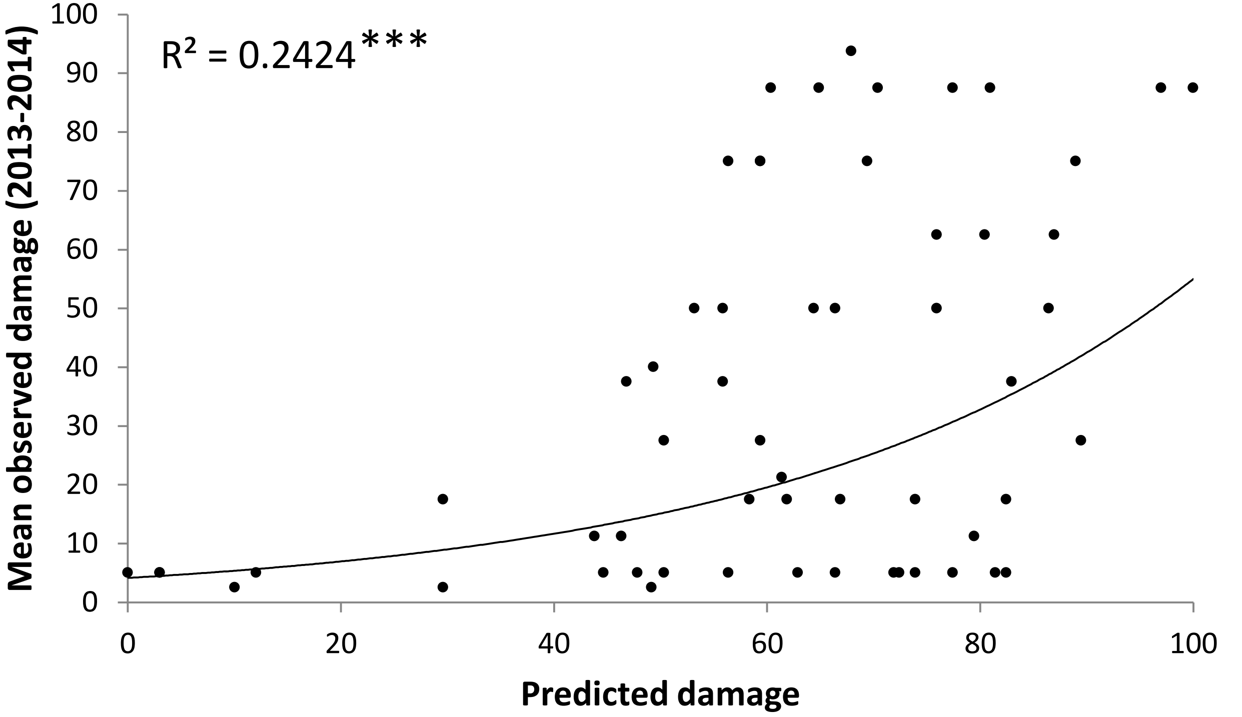

Supplementary Figure 4. Predicted vs observed damage scores for 66 *F. excelsior* accessions. Damage prediction was based on a re-scaled mean of the standardized prediction rank scores for the two individual GEM, and single cSNP prediction. * P<0.05, ** P<0.01, *** P<0.001.

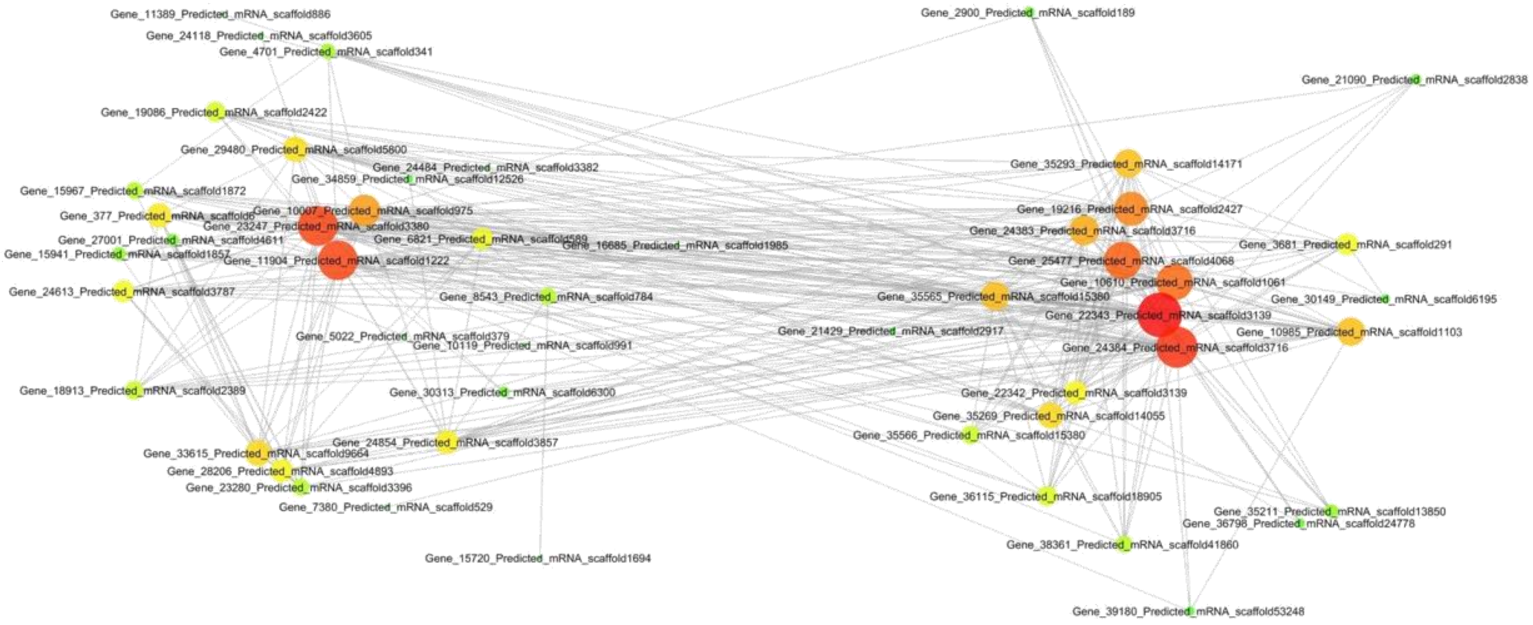

Supplementary Figure 5. Co-expression network. WGCNA identified a module containing 56 genes that was significantly associated with disease tolerance. Nodes are sized and coloured depending based on degree with the most connected "hub" genes shown as larger, redder nodes. Genes on the left side of the network show a positive correlation with canopy damage, and those on the right, a negative correlation.
